# Convergence rate for the moving least-squares learning with dependent sampling

**DOI:** 10.1186/s13660-018-1794-8

**Published:** 2018-07-31

**Authors:** Qin Guo, Peixin Ye

**Affiliations:** School of Mathematical Sciences and LPMC, Nankai University, Tianjin, China

**Keywords:** 68T05, 68Q32, 60E15, Moving least-squares, Regression function, Mixing sequence, Probability inequality, Error bound

## Abstract

We consider the moving least-squares (MLS) method by the regression learning framework under the assumption that the sampling process satisfies the *α*-mixing condition. We conduct the rigorous error analysis by using the probability inequalities for the dependent samples in the error estimates. When the dependent samples satisfy an exponential *α*-mixing, we derive the satisfactory learning rate and error bound of the algorithm.

## Introduction

The least-squares (LS) method is an important global approximate method based on the regular or concentrated data sample points. However, there are still some irregular or scattered samples which are obtained in many practical applications such as engineering and machine learning [1–4]. They also need to be analyzed to achieve their special usefulness. The moving least-squares (MLS) method was introduced by McLain in [4] to draw a set of contours based on a cluster of scattered data sample points. It turns out that the MLS method is a useful local approximation tool in various fields of mathematics such as approximation theory, data smoothing [5], statistics [6], and numerical analysis [7]. Recently, research effort has been made to study the regression learning algorithm by the MLS method, see [8–12]. The main advantage of the MLS regression learning algorithm is that we can learn the regression function in the simple function space, usually generated by polynomials.

We recall the regression learning problem by the MLS method briefly. Functions for learning are defined on a compact metric space *X* (input space) and take values in $Y=\mathbb{R}$ (output space). The sampling process is controlled by an unknown Borel probability measure *ρ* on $Z= X\times Y$. We define the regression function as follows:

$$f_{\rho}(x)= \int_{Y} y\, d\rho(y|x), $$

where $\rho(\cdot|x)$ is the conditional probability measure induced by *ρ* on *Y* given $x\in X$. The goal of regression learning is to find a good approximation of the regression function $f_{\rho}$ based on a set of random samples $\mathbf{z}=\{z_{i}\}_{i=1}^{m}=\{(x_{i}, y_{i})\}_{i=1}^{m} \in Z^{m}$ drawn according to the measure *ρ*.

We define the approximation $f_{\mathbf{z}}$ of $f_{\rho}$ pointwisely:

1.1 $$ f_{\mathbf{z}}(x):=f_{\mathbf{z},\sigma,x}(x)=\arg\min _{f\in \mathcal{H}}\mathcal{E}_{\mathbf{z},x}(f),\quad x\in X, $$

the local moving empirical error is defined by

1.2 $$ \mathcal{E}_{\mathbf{z},x}(f)=\frac{1}{m}\sum _{i=1}^{m}\Phi \biggl(\frac{x}{\sigma}, \frac{x_{i}}{\sigma} \biggr) \bigl(f(x_{i})-y_{i} \bigr)^{2}, $$

where the hypothesis space $\mathcal{H}\subseteq C(X)$ is a *d̃*-dimensional Lipschitz function space, $\sigma=\sigma(m)>0$ is a window width, and $\Phi:\mathbb{R}^{n}\times\mathbb{R}^{n}\to \mathbb{R}^{+}$ is called an MLS weight function which satisfies the conditions as follows, see [9, 10]:

1.3 $$\begin{aligned} (1)&\quad \int_{\mathbb{R}^{n}}\Phi (x,t)\,dt=1,\quad \forall x,t \in \mathbb{R}^{n}, \end{aligned}$$

1.4 $$\begin{aligned} (2)&\quad \Phi(x,t)\geq c_{q},\quad \forall \vert x-t \vert \leq1, \end{aligned}$$

1.5 $$\begin{aligned} (3)&\quad \bigl\vert \Phi(x,t) \bigr\vert \leq\frac{\tilde{c_{q}}}{(1+ \vert x-t \vert )^{q}},\quad \forall x,t\in\mathbb{R}^{n}, \end{aligned}$$

where the constants $q>n+1$, $c_{q}, \tilde{c_{q}}>0$.

The task of the paper is to derive the error bound of $\|f_{\mathbf {z}}-f_{\rho}\|_{\rho_{X}}$ with the norm $\|f(\cdot)\|_{\rho_{X}}:=(\int_{X}|f(\cdot)|^{2}\, d{\rho _{X}})^{\frac{1}{2}}$ to evaluate the approximation ability of $f_{\mathbf{z}}$, see [13–22]. The error analysis of algorithm (1.1) for the independent and identical (i.i.d.) samples has been carried out in [8–10]. However, the samples are not independent but are not far from being independent in some real data analysis such as market prediction, system diagnosis, and speech recognition. The mixing conditions can quantify how close to independence a sequence of random samples is. In [14, 16, 23–25], the authors carried out the regression estimation of the least squares algorithm with the *α*-mixing samples. Up to now there has been no result of algorithm (1.1) obtained in the case of dependent samples. Hence we extend the analysis of algorithm (1.1) to the *α*-mixing sampling setting which is quite easy to establish, see [26].

### Definition 1.1

Let $\mathcal {M}_{a}^{b} $ denote the *σ*-algebras of events generated by the random samples $\{z_{i}=(x_{i}, y_{i})\}_{i=a}^{b}$. $\{z_{i}\}_{i\geq1}$ is said to satisfy a strongly mixing condition (or *α*-mixing condition) if

1.6 $$ \alpha_{i}= \sup_{k\ge1} \sup_{A\in\mathcal {M}_{1}^{k},B\in\mathcal{M}_{k+i}^{\infty}} \bigl\vert P(A \cap B)-P(A)P(B) \bigr\vert \longrightarrow0,\quad \mbox{as } i\to\infty. $$

Specifically, if there exist some positive constants $\overline{\alpha }>0$, $\beta>0$, and $c>0$ such that

1.7 $$ \alpha_{i}\leq\overline{\alpha }\exp\bigl(-ci^{\beta} \bigr),\quad \forall i\geq1, $$

then it is said to satisfy an exponential strongly mixing condition.

Our goal is to obtain the convergence rate as $m \to\infty$ of algorithm (1.1) under hypothesis (1.7). The rest of the paper is organized as follows. In Sect. 2, we review some concepts and state our main results and the error decomposition. In Sect. 3, we present the estimate of the sample error. In Sect. 4, we provide the proofs of the main results.

## Main results and error decomposition

Before giving the main results, we firstly need to provide some concepts that will be referred to throughout this paper, see [8–10].

### Definition 2.1

The probability measure $\rho_{X} $ on *X* is said to satisfy the condition $L_{\tau}$ with exponent $\tau>0$ if

2.1 $$ \rho_{X} \bigl(B(x,r)\bigr)\geq c_{\tau}r^{\tau}, \quad \forall 0< r\leq r_{0}, x\in X, $$

where the constants $r_{0}>0$, $c_{\tau}>0$, and $B(x,r)=\{u\in X: |u-x|\leq r, \mbox{for }r>0\}$.

### Definition 2.2

We say that the hypothesis space $\mathcal{H}$ satisfies the norming condition with exponent $\zeta>0$ and $d\in\mathbb{N}$ if we can find points $\{u_{i}\}_{i=1}^{d}\subset B(x,\sigma)$ for every $x\in X$ and $0<\sigma\leq\sigma_{0}$ satisfying $|u_{i}-u_{j}|\geq 2c_{\mathcal{H}}\sigma$ for $i\neq j$ and

2.2 $$ \Biggl(\sum_{i=1}^{d} \bigl\vert f(u_{i}) \bigr\vert ^{2} \Biggr)^{\frac{1}{2}}\geq c_{\mathcal{H}}\sigma^{\zeta}\|f\|_{C(X)},\quad \forall f\in \mathcal{H}, $$

where the constants $\sigma_{0}>0$, $c_{\mathcal{H}}>0$ and *d* is chosen as at least the dimension *d̃* of $\mathcal{H}$.

Here we assume $|y|\leq M$ almost surely, and all the constants such as *C̃*, $C_{\mathcal{H},\zeta}$, $A_{\tau,\zeta}$, $C_{\mathcal{H},\rho _{X}}$, $C'_{\mathcal{H},\rho_{X}}$, and so on are independent of the key parameters *δ*, *m*, or *σ* in this paper. Now we give our main results of algorithm (1.1).

### Theorem 2.1

*Assume that* (1.7), (2.1), *and* (2.2) *hold*. *Suppose* $0< p<2$, $\sigma= (m^{(\alpha)} )^{-\gamma}$ *with* $m^{(\alpha)}= \lfloor m \lceil \{\frac{8m}{c} \} ^{1/(1+\beta)} \rceil^{-1} \rfloor$, $\gamma>0$, *and* $0<\sigma\leq\min \{\sigma_{0},1,(r_{0}/C_{\mathcal{H},\zeta })^{1/\max\{\zeta,1\}} \}$. *If* *m* *satisfies*

2.3 $$\begin{aligned} \bigl(m^{(\alpha)} \bigr)^{1-\gamma\tau\max\{\zeta,1\}} \geq& \bigl(256c_{p}^{-\frac{1}{p}}/3+A_{\tau,\zeta} \bigr) \bigl(\log \bigl(2+8e^{-2}\overline{\alpha}\bigr)/\delta \bigr)^{1+\frac{1}{p}} \\ &{}+A_{\tau,\zeta}\gamma\log m^{(\alpha)}, \end{aligned}$$

*then for any* $0<\delta<1$, *with confidence* $1-\delta$, *we have*

2.4 $$ \|f_{\mathbf{z}}-f_{\rho}\|_{\rho_{X}}^{2} \leq\widetilde{C} \bigl(m^{(\alpha)} \bigr)^{4\gamma(\zeta+\frac{\tau}{2}\max\{\zeta,1\} )-n\gamma-\frac{1}{p+1}}. $$

Then we can obtain the explicit learning rate of algorithm (1.1) with selecting the suitable parameter $\sigma=\sigma(m)$.

### Theorem 2.2

*Under the assumptions of Theorem *2.1, *if we choose* $\sigma= (m^{(\alpha)} )^{\frac{\varepsilon}{-(4\varsigma+2\max\{\tau,\tau\varsigma\} )}}$, $0<\varepsilon<1/4$, *and*

2.5 $$ m^{(\alpha)}\geq C_{1} \bigl(\bigl(\log \bigl(2+8e^{-2}\overline{\alpha }\bigr)/\delta\bigr)^{1+\frac{1}{p}}+\log m^{(\alpha)} \bigr)^{2}+\sigma _{0}^{-(4\varsigma+2\max\{\tau,\tau\varsigma\})/\varepsilon}, $$

*then with confidence* $1-\delta$, *we have*

2.6 $$ \|f_{\mathbf{z}}-f_{\rho}\|_{\rho_{X}}\leq C_{2} \bigl(m^{(\alpha )} \bigr)^{\varepsilon-\frac{1}{2}}, $$

*where*

2.7 $$ C_{1}= \biggl(A_{\tau,\zeta}+\frac{256}{3}c_{p}^{-\frac{1}{p}} \biggr)^{2} \biggl(1+\frac{1}{4\varsigma+2\max\{\tau,\tau\zeta\}} \biggr)^{2}. $$

### Remark 2.1

The result of the above theorem shows that the learning rate tends to $m^{-\frac{1}{2}}$ when $\sigma\rightarrow1$. For the i.i.d. case, the same rate has been obtained in [9, 10].

To estimate the quantity of the total error $\|f_{\mathbf{z}}-f_{\rho }\|_{\rho_{X}}$, we use the proposition from [8] below.

### Proposition 2.1

*Assume* (2.1) *and* (2.2) *hold*. *Then we have*

2.8 $$ \|f_{\mathbf{z}}-f_{\mathcal{H}}\|_{\rho_{X}}^{2} \leq\widetilde {C_{\mathcal{H}}}\sigma^{-2\zeta-\tau\max\{\zeta,1\}} \int_{X} \bigl(\mathcal{E}_{x}(f_{\mathbf{z},\sigma,x})- \mathcal {E}_{x}(f_{\mathcal{H},\sigma,x}) \bigr)\, d\rho_{X}(x), $$

*where*

2.9 $$ \mathcal{E}_{x}(f)= \int_{Z}\Phi \biggl(\frac{x}{\sigma},\frac {u}{\sigma} \biggr) \bigl(f(u)-y\bigr)^{2}\, d\rho(u,y),\quad \forall f: X \rightarrow \mathbb{R} $$

*is called the local moving expected risk and*

2.10 $$ f_{\mathcal{H}}(x):=f_{\mathcal{H},\sigma,x}=\arg\min _{f\in \mathcal{H}}\mathcal{E}_{x}(f),\quad x\in X, $$

*is called the target function*.

### Remark 2.2

Here we assume $f_{\rho}\in\mathcal{H}$. It follows from

2.11 $$ \mathcal{E}_{x}(f)= \int_{X}\Phi \biggl(\frac{x}{\sigma},\frac {u}{\sigma} \biggr) \bigl(f(u)-f_{\rho}(u)\bigr)^{2}\, d \rho_{X}(u)+\mathcal {E}_{x}(f_{\rho}),\quad \forall f: X\rightarrow\mathbb{R}, $$

that $f_{\mathcal{H}}=f_{\rho}$. Thus $\|f_{\mathbf{z}}-f_{\rho}\| _{\rho_{X}}=\|f_{\mathbf{z}}-f_{\mathcal{H}}\|_{\rho_{X}}$.

Next we only need to provide the upper bound of the integral in (2.8). So to do this, we give its decomposition as follows:

2.12 $$\begin{aligned} \int_{X} \bigl(\mathcal{E}_{x}(f_{\mathbf{z},\sigma,x})- \mathcal {E}_{x}(f_{\mathcal{H},\sigma,x}) \bigr)\,d\rho_{X}(x) \leq& \int _{X} \bigl[ \bigl(\mathcal{E}_{x}(f_{\mathbf{z},\sigma,x})- \mathcal {E}_{x}(f_{\mathcal{H},\sigma,x}) \bigr) \\ &{} - \bigl(\mathcal{E}_{\mathbf{z},x}(f_{\mathbf{z},\sigma ,x})-\mathcal{E}_{\mathbf{z},x}(f_{\mathcal{H},\sigma,x}) \bigr) \bigr]\,d\rho_{X}(x) \\ :=&\mathcal{S}(\mathbf{z},\sigma). \end{aligned}$$

What is left is to estimate the sample error $\mathcal{S}(\mathbf {z},\sigma)$.

## Estimates for the sample error

In order to obtain the probability estimate of $\mathcal{S}(\mathbf {z},\sigma)$, we shall use the upper bound for $f_{\mathbf{z},\sigma ,x}$ and $f_{\mathcal{H},\sigma,x}$. We firstly derive the confidence-based estimate of $f_{\mathbf {z},\sigma,x}$ as follows.

### Proposition 3.1

*Under the assumptions of Theorem *2.1, *if*

3.1 $$ m^{(\alpha)}\geq-A_{\tau,\zeta}\log(\delta\sigma) \sigma^{-\tau \max\{\zeta,1\}}, $$

*then with confidence at least* $1-\delta$, *we have*

3.2 $$ \|f_{\mathbf{z},\sigma,x}\|_{C(X)}\leq\frac{2^{3+\tau+\zeta }M}{\sqrt{c_{\tau}c_{q}}C_{\mathcal{H},\zeta}^{\tau/2}c_{\mathcal {H}}} \sigma^{-\zeta-\max\{\frac{\tau}{2},\frac{\tau\zeta}{2}\} }:=C_{\mathcal{H},\rho_{X}}\sigma^{-\zeta-\max\{\frac{\tau }{2},\frac{\tau\zeta}{2}\}},\quad \forall x\in X, $$

*where*

$$\begin{aligned}& C_{\mathcal{H},\zeta}=\min \biggl\{ \frac{c_{\mathcal{H}}}{2^{\zeta +1}\sqrt{d}C_{\mathcal{H},0}}, \frac{c_{\mathcal{H}}}{2}, \frac {1}{2} \biggr\} , \\& A_{\tau,\zeta}=2^{\tau+1}\bigl(c_{\tau}C_{\mathcal{H},\zeta}^{\tau }-2^{\tau+1} \bigr)^{-1} \biggl[\frac{7}{6}+\frac{7}{6}\log \bigl(1+4e^{-2}\overline{\alpha}\bigr)+\frac{7n}{6}\log \biggl(1+ \frac {4B_{X}}{C_{\mathcal{H},\zeta}} \biggr) \biggr]. \end{aligned}$$

The proof is analogous to that of Theorem 3 in [8] except that we need to use the following Lemma 3.1 for the dependent sampling setting to replace Lemma 2 in [8].

### Lemma 3.1

*Let* $0< r\leq r_{0}$ *and* $0<\delta<1$. *If* (1.7) *and* (2.1) *hold*, *then with confidence* $1-\delta$, *we have*

3.3 $$\begin{aligned} \frac{\sharp({\mathbf{x}}\cap B(x,r))}{m} \geq&c_{\tau} \biggl(\frac{r}{2} \biggr)^{\tau}+\frac{7\log\delta-7\log (1+4e^{-2}\overline{\alpha})-7n\log(\frac {4B_{X}}{r}+1)}{6m^{(\alpha)}} \\ &{}-1,\quad \forall x\in X. \end{aligned}$$

*Specifically*, *if*

3.4 $$ m^{(\alpha)}>\frac{ [7\log(\frac{1}{\delta})+7\log (1+4e^{-2}\overline{\alpha}) +7n\log(\frac{4B_{X}}{r}+1) ]}{6(\frac{c_{\tau}r^{\tau }}{2^{\tau+1}}-1)}, $$

*then with confidence at least* $1-\delta$, *we have*

3.5 $$ \frac{\sharp({\mathbf{x}}\cap B(x,r))}{m}\geq\frac{c_{\tau }}{2^{\tau+1}}r^{\tau},\quad \forall x\in X, $$

*where* $\frac{\sharp({\mathbf{x}}\cap B(x,r))}{m}$ *is the proportion of those sampling points lying in* $B(x,r)$.

### Proof

It is shown in Theorem 5.3 of [27] that one can find $\{v_{j}\}_{j=1}^{\mathcal{N}}\subseteq X$ satisfying $X\subseteq B_{R}(\mathbb{R}^{n})\subseteq\bigcup_{j=1}^{\mathcal {N}}B(v_{j},\frac{r}{2})$ and $\mathcal{N}\leq(\frac{4R}{r}+1)^{n}$. Let $\xi^{(j)}: X\rightarrow\mathbb{R}$ be the characteristic function of the set $B(v_{j},\frac{r}{2})$. Its mean $\mu^{(j)}=\int _{X} \xi^{(j)}(x)\,d\rho_{X}=\rho_{X}(B(v_{j},\frac{r}{2}))$ satisfies $|\xi^{(j)}-\mu^{(j)}|\leq1$ and $\sigma^{2}(\xi ^{(j)})\leq1$. Now we use the Bernstein inequality for the dependent samples in [28].

### Proposition 3.2

*Suppose that* (1.7) *holds*. *Let the random variable* $m^{(\alpha)}$ *be the effective number of observations and* $\xi _{i}=\xi(z_{i})$ *be a real*-*valued function on the probability space* *Z* *with mean* $\mu=\int_{Z} \xi(z)\,d\rho$ *and variance* $\sigma ^{2}$. *Assume that* $|\xi_{i}-\mu|\leq D$ *almost surely*. *Then*, *for every* $\varepsilon>0$,

3.6 $$ P \Biggl\{ \frac{1}{m}\sum_{i=1}^{m} [\xi_{i}-\mu ]>\varepsilon \Biggr\} \leq\bigl(1+4e^{-2} \overline{\alpha}\bigr)\exp \biggl\{ -\frac{m^{(\alpha)}\varepsilon^{2}}{2(\sigma^{2}+\frac {1}{3}D\varepsilon)} \biggr\} . $$

Then it follows from the above proposition that

3.7 $$ P \Biggl\{ \frac{1}{m}\sum_{i=1}^{m} \bigl[\xi^{(j)}_{i}-\mu^{(j)} \bigr]\leq-\varepsilon \Biggr\} \leq\bigl(1+4e^{-2}\overline{\alpha}\bigr)\exp \biggl\{ - \frac{m^{(\alpha)}\varepsilon^{2}}{2+\frac {2}{3}\varepsilon} \biggr\} ,\quad \forall\varepsilon>0, $$

hence,

3.8 $$ P \Biggl\{ \min_{1\leq j\leq\mathcal{N}} \Biggl\{ \frac{1}{m}\sum _{i=1}^{m} \bigl[\xi^{(j)}_{i}- \mu^{(j)} \bigr] \Biggr\} \leq -\varepsilon \Biggr\} \leq\mathcal{N} \bigl(1+4e^{-2}\overline{\alpha }\bigr)\exp \biggl\{ -\frac{m^{(\alpha)}\varepsilon^{2}}{2+\frac {2}{3}\varepsilon} \biggr\} . $$

For $0<\delta<1$, let

3.9 $$ \mathcal{N}\bigl(1+4e^{-2}\overline{\alpha}\bigr)\exp \biggl\{ - \frac {m^{(\alpha)}\varepsilon^{2}}{2+\frac{2}{3}\varepsilon} \biggr\} =\delta. $$

Then we get

3.10 $$\begin{aligned} \varepsilon&= \frac{\frac{2}{3}\log\frac{\mathcal{N}(1+4e^{-2}\overline{\alpha })}{\delta}+\sqrt{ (\frac{2}{3}\log\frac{\mathcal {N}(1+4e^{-2}\overline{\alpha})}{\delta} )^{2}+8m^{(\alpha )}\log\frac{\mathcal{N}(1+4e^{-2}\overline{\alpha})}{\delta }}}{2m^{(\alpha)}} \\ &\leq\frac{2\log\frac{\mathcal{N}(1+4e^{-2}\overline{\alpha })}{\delta}}{3m^{(\alpha)}}+\sqrt{\frac{2\log\frac{\mathcal {N}(1+4e^{-2}\overline{\alpha})}{\delta}}{m^{(\alpha)}}} \\ &\leq\frac{7\log\frac{\mathcal{N}(1+4e^{-2}\overline{\alpha })}{\delta}}{6m^{(\alpha)}}+1. \end{aligned}$$

It follows that, with confidence at least $1-\delta$,

3.11 $$ \min_{1\leq j\leq\mathcal{N}} \Biggl\{ \frac{1}{m}\sum _{i=1}^{m} \bigl[\xi^{(j)}_{i}- \mu^{(j)} \bigr] \Biggr\} >-\frac{7\log\frac{\mathcal {N}(1+4e^{-2}\overline{\alpha})}{\delta}}{6m^{(\alpha)}}-1. $$

Hence, we have

3.12 $$ \frac{1}{m}\sum_{i=1}^{m} \bigl[\xi^{(j)}-\mu^{(j)} \bigr]>-\frac {7\log\frac{\mathcal{N}(1+4e^{-2}\overline{\alpha})}{\delta }}{6m^{(\alpha)}}-1,\quad \forall j=1,\ldots,\mathcal{N}. $$

Condition (2.1) yields $\mu^{(j)}\geq c_{\tau} (\frac {r}{2} )^{\tau}$. Also $\xi^{(j)}(x_{i})=1$ if $x_{i}\in B(v_{j},\frac{r}{2})$ and 0 otherwise. So that $\frac{1}{m}\sum_{i=1}^{m}\xi^{(j)}(x_{i})=\sharp ({\mathbf{x}}\cap B(v_{j},\frac{r}{2}))/m$. Hence,

3.13 $$ \sharp\biggl({\mathbf{x}}\cap B\biggl(v_{j},\frac{r}{2}\biggr) \biggr)\big/m>c_{\tau} \biggl(\frac{r}{2} \biggr)^{\tau}- \frac{7\log\frac{\mathcal {N}(1+4e^{-2}\overline{\alpha})}{\delta}}{6m^{(\alpha)}}-1,\quad \forall j=1,\ldots,\mathcal{N}. $$

Observe from $X\subseteq\bigcup_{j=1}^{\mathcal{N}}B(v_{j},\frac {r}{2})$ that for each $x\in X$, there exists some $j\in {1,\ldots,\mathcal{N}}$ such that $x\in B(v_{j},\frac{r}{2})$, i.e., $|v_{j}-x|\leq\frac{r}{2}$. Since $x_{i}\in B(v_{j},\frac{r}{2})$ implies $|x_{i}-x|\leq|x_{i}-v_{j}|+|v_{j}-x|\leq r$, we see that

3.14 $$\begin{aligned} \sharp\bigl({\mathbf{x}}\cap B(x,r)\bigr)/m&\geq\sharp\biggl({\mathbf {x}}\cap B \biggl(v_{j},\frac{r}{2}\biggr)\biggr)\big/m \\ &\geq c_{\tau} \biggl(\frac{r}{2} \biggr)^{\tau}- \frac{7\log\frac {\mathcal{N}(1+4e^{-2}\overline{\alpha})}{\delta}}{6m^{(\alpha)}}-1. \end{aligned}$$

This proves Lemma 3.1.  □

Now we are in a position to prove Proposition 3.1.

### Proof of Proposition 3.1

By (3.1) and setting $r=C_{\mathcal{H},\zeta}\sigma^{\max\{ \zeta,1\}}\leq r_{0}$, it is easy to see that (3.4) holds. Then (3.5) is valid.

It follows from (3.5) and Definition 2.2 with *σ* replaced by $\frac{\sigma}{2}$ that

3.15 $$ m_{i}/m=\sharp\bigl({\mathbf{x}}\cap B(u_{i},r) \bigr)/m>c_{\tau}r^{\tau }/2^{\tau+1}, $$

and

3.16 $$ |x_{i,l}-u_{i}|\leq r, $$

where $\{x_{i,l}\}_{l=1}^{m_{i}}$ are the points of the set $\sharp ({\mathbf{x}}\cap B(u_{i},r))$, which implies

3.17 $$ |x_{i,l}-x|\leq|x_{i,l}-u_{i}|+|u_{i}-x| \leq r+\frac{\sigma}{2}\leq \sigma, $$

where $x\in X$, $l=1,\ldots,\tilde{m}$, and $\tilde {m}=\min_{1\leq i\leq d}\{m_{i}\}$.

Then, by (1.4), we have

3.18 $$ \biggl\vert \Phi\biggl(\frac{x}{\sigma},\frac{x_{i,l}}{\sigma}\biggr) \biggr\vert \geq c_{q}. $$

Hence

3.19 $$\begin{aligned} \frac{1}{m}\sum_{i=1}^{m}\Phi \biggl( \frac{x}{\sigma},\frac {x_{j}}{\sigma} \biggr) \bigl(f_{\mathbf{z},\sigma,x}(x_{j}) \bigr)^{2}&\geq\frac {1}{m}\sum_{i=1}^{d} \sum_{l=1}^{\widetilde{m}}\Phi \biggl( \frac {x}{\sigma},\frac{x_{i,l}}{\sigma} \biggr) \bigl(f_{\mathbf{z},\sigma ,x}(x_{i,l}) \bigr)^{2} \\ &\geq\frac{1}{m}\sum_{i=1}^{d}\sum _{l=1}^{\widetilde {m}}c_{q} \bigl(f_{\mathbf{z},\sigma,x}(x_{i,l})\bigr)^{2} \\ &\geq\frac{c_{\tau}}{2^{\tau+1}}r^{\tau}c_{q} \biggl(\frac {c_{\mathcal{H}}\sigma^{\zeta}}{2^{\zeta+1}} \biggr)^{2}\|f_{\mathbf {z},\sigma,x}\|_{C(X)}^{2}. \end{aligned}$$

The last inequality has been proved in Theorem 3 in [8].

Finally, combining (3.19) with the following inequality

3.20 $$ \frac{1}{m}\sum_{i=1}^{m} \Phi \biggl(\frac{x}{\sigma},\frac {x_{i}}{\sigma} \biggr) \bigl(f_{\mathbf{z},\sigma,x}(x_{i}) \bigr)^{2}\leq\frac {2}{m}\sum_{i=1}^{m} \Phi \biggl(\frac{x}{\sigma},\frac{x_{i}}{\sigma } \biggr) \bigl\{ (0-y_{i})^{2}+y_{i}^{2} \bigr\} \leq4M^{2}, $$

we derive the desired result. □

We also need to invoke Lemma 4 in [8] which provides the result about the upper bound of $f_{\mathcal{H},\sigma,x}$.

### Proposition 3.3

*Assume that* (2.1) *and* (2.2) *hold*. *Then*, *for some constant* $C'_{\mathcal{H},\rho _{X}}$ *independent of* *σ*, *we have*

3.21 $$ \|f_{\mathcal{H},\sigma,x}\|_{C(X)}\leq C'_{\mathcal{H},\rho _{X}} \sigma^{-\zeta-\max\{\frac{\tau}{2},\frac{\tau\zeta}{2}\} },\quad \forall x\in X, 0< \sigma\leq\min\{ \sigma_{0},1\}. $$

Next we will bound the sample error. The estimation for $\mathcal {S}(\mathbf{z},\sigma)$ relies on the ratio probability inequality below that can be found in [27].

### Proposition 3.4

*Suppose that* (1.7) *holds*. *Let* $\mathcal{G}$ *be a set of functions on* *Z* *and* $c>0$ *such that*, *for each* $g\in\mathcal {G}$, $\mu(g)=\int_{Z}g(z)\,d\rho\geq0$, $\mu(g^{2})\leq c\mu(g)$, *and* $|g(z)-\mu(g)|\leq D$ *almost surely*. *Then*, *for every* $\varepsilon >0$ *and* $0<\alpha\leq1$, *we have*

3.22 $$\begin{aligned} P \biggl\{ \sup_{g\in\mathcal{G}}\frac{\mu(g)-\frac{1}{m}\sum_{i=1}^{m}g(z_{i})}{\sqrt{\varepsilon+\mu(g)}}\geq4\alpha\sqrt { \varepsilon} \biggr\} &\leq\bigl(1+4e^{-2}\overline{\alpha} \bigr) \mathcal{N}(\mathcal{G},\alpha\varepsilon) \\ &\quad {}\times\exp \biggl\{ -\frac{\alpha^{2} m^{(\alpha)}\varepsilon }{2c+\frac{2}{3}D} \biggr\} . \end{aligned}$$

We obtain the upper bound estimate for $\mathcal{S}(\mathbf{z},\sigma )$ by using Proposition 3.4.

### Proposition 3.5

*If the assumptions of Proposition *3.1 *hold*,

3.23 $$ R=\max\bigl\{ C_{\mathcal{H},\rho_{X}}, C'_{\mathcal{H},\rho _{X}}, M\bigr\} \sigma^{-\zeta-\frac{\tau}{2}\max\{1,\zeta\})}, $$

*and*

3.24 $$ m^{(\alpha)}\geq\frac{256}{3}c_{p}^{-\frac{1}{p}} \biggl(\log \frac {2+8e^{-2}\overline{\alpha}}{\delta} \biggr)^{1+\frac{1}{p}}, $$

*then with confidence* $1-\delta$, *there holds*

3.25 $$\begin{aligned} & \int_{X} \bigl[ \bigl(\mathcal{E}_{x}(f_{\mathbf{z},\sigma ,x})- \mathcal{E}_{x}(f_{\mathcal{H},\sigma,x}) \bigr)- \bigl(\mathcal {E}_{\mathbf{z},x}(f_{\mathbf{z},\sigma,x})-\mathcal{E}_{\mathbf {z},x}(f_{\mathcal{H},\sigma,x}) \bigr) \bigr]\,d\rho_{X}(x) \\ &\quad \leq16 R^{2}D\sigma^{n} \biggl(\frac{256c_{p}}{3m^{(\alpha)}} \biggr)^{1/(1+p)}+\frac{1}{2} \int_{X} \bigl(\mathcal{E}_{x}(f_{\mathbf {z},\sigma,x})- \mathcal{E}_{x}(f_{\mathcal{H},\sigma,x}) \bigr)\,d\rho_{X}(x). \end{aligned}$$

### Proof

Let the function $g(u,y)$ be defined on the function set

3.26 $$\begin{aligned} \mathcal{G}_{R}&= \biggl\{ \int_{X}\Phi \biggl(\frac{x}{\sigma},\frac {u}{\sigma} \biggr)\bigl[\bigl(f(u)-y\bigr)^{2}-\bigl(f_{\mathcal{H},\sigma ,x}(u)-y \bigr)^{2}\bigr]\,d\rho_{X}(x): \\ &\quad {}f\in B_{R}:=\{f\in\mathcal{H}:\|f\|_{C(X)}\leq R\} \biggr\} . \end{aligned}$$

With condition (1.5) and the bound $c_{\rho}$ of the density function of $\rho_{X}$, we have

3.27 $$ \int_{X}\Phi \biggl(\frac{x}{\sigma},\frac{u}{\sigma} \biggr)\,d\rho _{X}(x)\leq c_{\rho}\widetilde{c_{q}} \int_{\mathbb{R}^{n}}\frac {\sigma^{n}}{(1+|u|)^{ q}}\,du\leq\frac{2\pi^{n/2}c_{\rho}\tilde {c_{q}}}{(q-n)\Gamma(\frac{n}{2})} \sigma^{n}:=D\sigma^{n}, $$

which implies

3.28 $$ \bigl\vert g(u,y) \bigr\vert \leq2(R+M)^{2}D\sigma^{n} \leq8R^{2}D\sigma^{n}:=c_{R}. $$

Hence $|g(u,y)-\mu(g)|\leq2c_{R}$.

It follows from the Schwarz inequality that

3.29 $$\begin{aligned} \bigl\vert g(u,y) \bigr\vert ^{2}&= \biggl\vert \int_{X}\Phi \biggl(\frac{x}{\sigma},\frac {u}{\sigma} \biggr) \bigl(f(u)-f_{\mathcal{H},\sigma,x}(u) \bigr) \bigl(f(u)+f_{\mathcal{H},\sigma,x}(u)-2y \bigr)\,d\rho_{X}(x) \biggr\vert ^{2} \\ &\leq \int_{X}\Phi \biggl(\frac{x}{\sigma},\frac{u}{\sigma} \biggr) \bigl(f(u)-f_{\mathcal{H},\sigma,x}(u) \bigr)^{2}(2R+2M)^{2} \,d\rho _{X}(x) \\ &\quad {} \times \int_{X}\Phi \biggl(\frac{x}{\sigma},\frac{u}{\sigma} \biggr)\,d\rho_{X}(x). \end{aligned}$$

By (3.27),

3.30 $$ \mu\bigl(g^{2}\bigr)\leq16R^{2}D \sigma^{n} \int_{X} \biggl( \int_{X}\Phi \biggl(\frac{x}{\sigma},\frac{u}{\sigma} \biggr) \bigl(f(u)-f_{\mathcal {H},\sigma,x}(u) \bigr)^{2}\,d \rho_{X}(u) \biggr)\,d\rho_{X}(x). $$

It has been proved in [9] that

3.31 $$\begin{aligned}& \int_{X}\Phi \biggl(\frac{x}{\sigma},\frac{u}{\sigma} \biggr) \bigl(f(u)-f_{\mathcal{H},\sigma,x}(u) \bigr)^{2}\,d \rho_{X}(u) \\& \quad = \int _{Z}\Phi \biggl(\frac{x}{\sigma},\frac{u}{\sigma} \biggr)\bigl[\bigl(f(u)-y\bigr)^{2} -\bigl(f_{\mathcal{H},\sigma,x}(u)-y\bigr)^{2}\bigr]\,d \rho(u,y). \end{aligned}$$

Substituting (3.31) into (3.30),

3.32 $$\begin{aligned} \mu\bigl(g^{2}\bigr)&\leq16R^{2}D\sigma^{n} \int_{X} \biggl( \int_{Z}\Phi \biggl(\frac{x}{\sigma},\frac{u}{\sigma} \biggr)\bigl[\bigl(f(u)-y\bigr)^{2} \\ &\quad {} -\bigl(f_{\mathcal{H},\sigma,x}(u)-y\bigr)^{2}\bigr]\,d\rho(u,y) \biggr)\,d\rho _{X}(x) \\ &=16R^{2}D\sigma^{n} \int_{Z} \biggl( \int_{X}\Phi \biggl(\frac {x}{\sigma},\frac{u}{\sigma} \biggr)\bigl[\bigl(f(u)-y\bigr)^{2} \\ &\quad {} -\bigl(f_{\mathcal{H},\sigma,x}(u)-y\bigr)^{2}\bigr]\,d \rho_{X}(x) \biggr)\,d\rho (u,y) \\ &=16R^{2}D\sigma^{n}\mu(g). \end{aligned}$$

Using Proposition 3.4 with $\alpha=\frac{1}{4}$ and $\mathcal{G}=\mathcal{G}_{R}$, we know that

3.33 $$\begin{aligned} &P \biggl\{ \sup_{f\in B_{R}}\frac{\int_{X} [ (\mathcal {E}_{x}(f)-\mathcal{E}_{x}(f_{\mathcal{H},\sigma,x}) )- (\mathcal{E}_{\mathbf{z},x}(f)-\mathcal{E}_{\mathbf {z},x}(f_{\mathcal{H},\sigma,x}) ) ]\,d\rho_{X}(x)}{\sqrt {\varepsilon+\int_{X} (\mathcal{E}_{x}(f)-\mathcal {E}_{x}(f_{\mathcal{H},\sigma,x}) )\,d\rho_{X}(x)}}\geq\sqrt { \varepsilon} \biggr\} \\ &\quad \leq\bigl(1+4e^{-2}\overline{\alpha} \bigr)\mathcal{N}\biggl( \mathcal{G}_{R},\frac{\varepsilon}{4}\biggr)\exp \biggl\{ - \frac{3m^{(\alpha)}\varepsilon}{2048R^{2}D\sigma^{n}} \biggr\} . \end{aligned}$$

Since for any $g_{1}, g_{1}\in\mathcal{G}_{R}$,

3.34 $$\begin{aligned} \bigl\vert g_{1}(u,y)-g_{2}(u,y) \bigr\vert &= \biggl\vert \int_{X}\Phi \biggl(\frac{x}{\sigma },\frac{u}{\sigma} \biggr) \bigl(\bigl(f_{1}(u)-y\bigr)^{2}- \bigl(f_{2}(u)-y\bigr)^{2} \bigr)\,d\rho_{X}(x) \biggr\vert \\ &\leq \biggl\vert \int_{X}\Phi \biggl(\frac{x}{\sigma},\frac{u}{\sigma } \biggr) \bigl(f_{1}(u)-f_{2}(u)\bigr) \\ &\quad {} \times\bigl(f_{1}(u)+f_{2}(u)-2y\bigr)\,d \rho_{X}(x) \biggr\vert \\ &\leq4 R D\sigma^{n} \bigl\vert f_{1}(u)-f_{2}(u) \bigr\vert , \end{aligned}$$

then we have

3.35 $$\begin{aligned} \mathcal{N} \biggl(\mathcal{G}_{R},\frac{\varepsilon}{4} \biggr)&\leq \mathcal{N} \biggl(B_{R},\frac{\varepsilon}{16 R D\sigma^{n}} \biggr) \\ &=\mathcal{N} \biggl(B_{1},\frac{\varepsilon}{16R^{2}D\sigma ^{n}} \biggr). \end{aligned}$$

It follows from (3.33) that

3.36 $$\begin{aligned} &P \biggl\{ \sup_{f\in B_{R}}\frac{\int_{X} [ (\mathcal {E}_{x}(f)-\mathcal{E}_{x}(f_{\mathcal{H},\sigma,x}) )- (\mathcal{E}_{\mathbf{z},x}(f)-\mathcal{E}_{\mathbf {z},x}(f_{\mathcal{H},\sigma,x}) ) ]\,d\rho_{X}(x)}{\sqrt {\varepsilon+\int_{X} (\mathcal{E}_{x}(f)-\mathcal {E}_{x}(f_{\mathcal{H},\sigma,x})\,d\rho_{X}(x)}}\leq\sqrt { \varepsilon} \biggr\} \\ &\quad \geq1-\bigl(1+4e^{-2}\overline{\alpha} \bigr)\mathcal{N} \biggl(B_{1},\frac{\varepsilon}{16R^{2}D\sigma ^{n}} \biggr)\exp \biggl\{ -\frac{3m^{(\alpha)}\varepsilon }{2048R^{2}D\sigma^{n}} \biggr\} . \end{aligned}$$

We set the term $(1+4e^{-2}\overline{\alpha} )\mathcal{N} (B_{1},\frac{\varepsilon}{16R^{2}D\sigma^{n}} )\exp \{-\frac{3m^{(\alpha)}\varepsilon}{2048R^{2}D\sigma ^{n}} \}$ of the above inequality to $\delta/2$. We need to invoke the lemma proved by the same method of Proposition 4.3 in [21].

### Lemma 3.2

*Let* $\eta^{\ast}(m^{(\alpha)},\delta)$ *be the smallest positive solution of the following inequality in η*:

$$\bigl(1+4e^{-2}\overline{\alpha} \bigr)\mathcal{N}(B_{1}, \eta))\exp\biggl\{ -\frac{3m^{(\alpha)}\eta}{128}\biggr\} \leq\delta. $$

*If* $\log\mathcal{N}(B_{1},\eta)\leq c_{p}(\eta)^{-p}$, *for some* $p\in(0,2)$, $c_{p}>0$ *and all* $\eta>0$, *then with confidence at least* $1-\delta$, *we have*

3.37 $$ \eta^{\ast}\bigl(m^{(\alpha)},\delta\bigr)\leq\max \biggl\{ \frac {256}{3m^{(\alpha)}}\log\frac{1+4e^{-2}\overline{\alpha}}{\delta }, \biggl(\frac{256c_{p}}{3m^{(\alpha)}} \biggr)^{1/(1+p)} \biggr\} . $$

Then we return to the proof of Proposition 3.5.

It follows from Theorem 5.3 in [27] that

3.38 $$ \mathcal{N} \biggl(B_{1},\frac{\varepsilon}{16R^{2}D\sigma^{n}} \biggr) \textstyle\begin{cases} \leq (\frac{32 R^{2}D\sigma^{n}}{\varepsilon}+1 )^{\tilde {d}}, & \mbox{for } 0< \varepsilon< 16 R^{2}D\sigma^{n}; \\ =1, & \mbox{for } \varepsilon\geq16 R^{2}D\sigma^{n}. \end{cases} $$

When $0<\varepsilon<16 R^{2}D\sigma^{n}$,

3.39 $$\begin{aligned} \begin{aligned}[b] \log\mathcal{N} \biggl(B_{1},\frac{\varepsilon}{16R^{2}D\sigma ^{n}} \biggr)&\leq\tilde{d} \log \biggl(\frac{32 R^{2}D\sigma ^{n}}{\varepsilon}+1 \biggr) \\ &\leq\frac{\tilde{d}}{p} \biggl(\frac{32 R^{2}D\sigma ^{n}}{\varepsilon} \biggr)^{p} \\ &=\frac{\tilde{d}}{p}\bigl(32 R^{2}D\sigma^{n} \bigr)^{p}\varepsilon^{-p},\quad p>0. \end{aligned} \end{aligned}$$

When $\varepsilon\geq16 R^{2}D\sigma^{n}$, we have

3.40 $$ \log\mathcal{N} \biggl(\mathcal{G}_{R},\frac{\varepsilon}{4} \biggr) \leq0. $$

Hence we conclude that

3.41 $$ \log\mathcal{N} \biggl(B_{1},\frac{\varepsilon}{16R^{2}D\sigma ^{n}} \biggr) \leq\frac{2^{p}\tilde{d}}{p} \biggl(\frac{\varepsilon }{16 R^{2}D\sigma^{n}} \biggr)^{-p}:=c_{p} \eta^{-p}. $$

This, together with (3.37), implies that, for

3.42 $$ m^{(\alpha)}\geq\frac{256}{3}c_{p}^{-\frac {1}{p}} \biggl(\log\frac{2+8e^{-2}\overline{\alpha}}{\delta} \biggr)^{1+\frac{1}{p}}, $$

we obtain

3.43 $$ \varepsilon\leq16 R^{2}D\sigma^{n} \biggl( \frac {256c_{p}}{3m^{(\alpha)}} \biggr)^{1/(1+p)}. $$

Combining (3.43) with (3.36), with confidence $1-\delta /2$, we have

3.44 $$\begin{aligned} & \int_{X} \bigl[ \bigl(\mathcal{E}_{x}(f)- \mathcal{E}_{x}(f_{\mathcal {H},\sigma,x}) \bigr)- \bigl(\mathcal{E}_{\mathbf{z},x}(f)- \mathcal {E}_{\mathbf{z},x}(f_{\mathcal{H},\sigma,x}) \bigr) \bigr]\,d\rho _{X}(x) \\ &\quad \leq\sqrt{\varepsilon}\sqrt{\varepsilon+ \int_{X} \big(\mathcal {E}_{x}(f)-\mathcal{E}_{x}(f_{\mathcal{H},\sigma,x})\big) \,d\rho_{X}(x)} \\ &\quad \leq\varepsilon+\frac{1}{2} \int_{X} \big(\mathcal {E}_{x}(f)-\mathcal{E}_{x}(f_{\mathcal{H},\sigma,x})\big) \,d\rho _{X}(x) \\ &\quad \leq16 R^{2}D\sigma^{n} \biggl(\frac{256c_{p}}{3m^{(\alpha)}} \biggr)^{1/(1+p)}+\frac{1}{2} \int_{X} \bigl(\mathcal{E}_{x}(f)-\mathcal {E}_{x}(f_{\mathcal{H},\sigma,x}) \bigr)\,d\rho_{X}(x). \end{aligned}$$

Finally, setting $f=f_{\mathbf{z},\sigma,x}$ in the above inequality, we derive the desired result.  □

## Proofs of the main results

In this subsection, we provide the proofs of Theorem 2.1 and Theorem 2.2. We firstly prove Theorem 2.1.

### Proof

If we take $\sigma= (m^{(\alpha)} )^{-\gamma}$, $\gamma>0$, then we have

4.1 $$ R=\max\bigl\{ C_{\mathcal{H},\rho_{X}}, C'_{\mathcal{H},\rho_{X}}, M\bigr\} m^{\gamma(\zeta+\frac{\tau}{2}\max\{1,\zeta\})}. $$

It is readily seen that (2.3) implies (3.1). Then Proposition 3.5 holds true. We thus obtain, with confidence $1-\delta$,

4.2 $$ \int_{X} \bigl(\mathcal{E}_{x}(f_{\mathbf{z},\sigma,x})- \mathcal {E}_{x}(f_{\mathcal{H},\sigma,x}) \bigr)\,d\rho_{X}(x) \leq32 R^{2}D\sigma^{n} \biggl(\frac{256c_{p}}{3m^{(\alpha)}} \biggr)^{1/(1+p)}. $$

Therefore from (2.8) we obtain

4.3 $$\begin{aligned} \|f_{\mathbf{z}}-f_{\mathcal{H}}\|_{\rho_{X}}^{2}&\leq \widetilde {C_{\mathcal{H}}}\sigma^{-2\zeta-\tau\max\{\zeta,1\}}\times32 R^{2}D \sigma^{n} \biggl(\frac{256c_{p}}{3m^{(\alpha)}} \biggr)^{1/(1+p)} \\ &\leq\widetilde{C}\bigl(m^{(\alpha)}\bigr)^{4\gamma(\zeta+\frac{\tau }{2}\max\{\zeta,1\})-n\gamma-\frac{1}{p+1}}. \end{aligned}$$

This proves Theorem 2.1. □

Next, we prove Theorem 2.2.

### Proof

Let $\gamma=\varepsilon/[4\varsigma+2\max\{\tau,\tau\varsigma\} ]>0$ and $p=\frac{\varepsilon}{1-\varepsilon}$. Therefore we have

4.4 $$ \gamma\tau\max\{1,\varsigma\}< \varepsilon< 1/4 $$

and

4.5 $$ \bigl(m^{(\alpha)}\bigr)^{1-\gamma\tau\max\{1,\varsigma\}}\geq\bigl(m^{(\alpha )} \bigr)^{1-\varepsilon}\geq m^{\frac{1}{2}}. $$

It follows from (2.5) that

4.6 $$\begin{aligned} \bigl(m^{(\alpha)}\bigr)^{\frac{1}{2}}&\geq \biggl(A_{\tau,\zeta}+ \frac {256}{3}c_{p}^{-\frac{1}{p}} \biggr) \biggl(1+ \frac{1}{4\varsigma +2\max\{\tau,\tau\zeta\}} \biggr) \\ &\quad {} \times \Bigl(\bigl(\log\bigl(2+8e^{-2}\overline{\alpha}\bigr)/ \delta\bigr)^{1+\frac {1}{p}}+\log m^{(\alpha)}\Bigr) \\ &\geq \biggl(A_{\tau,\zeta}+\frac{256}{3}c_{p}^{-\frac{1}{p}} \biggr) \Bigl(\bigl(\log\bigl(2+8e^{-2}\overline{\alpha}\bigr)/\delta \bigr)^{1+\frac {1}{p}}+\gamma\log m^{(\alpha)} \Bigr), \end{aligned}$$

which implies that condition (2.3) of Theorem 2.1 holds true, we thus obtain, with confidence $1-\delta$,

4.7 $$ \|f_{\mathbf{z}}-f_{\mathcal{H}}\|_{\rho_{X}}\leq C_{1} \bigl(m^{(\alpha)} \bigr)^{\varepsilon-\frac{1}{2}}. $$

This proves Theorem 2.2. □
